# Supplementary material for: Efficacy of Chemotherapy in Pain Control of Patients with Cancer at the Early Phase of Their Disease
Source: Healthcare (Basel). 2025 Apr 18;13(8):931. doi: 10.3390/healthcare13080931 (PMC12027393; doi:10.3390/healthcare13080931)
Supplement: Supplementary file 1 [file healthcare-13-00931-s001.zip › healthcare-3506356-supplementary.pdf]

Supplementary Materials

To check whether the reduction in pain is significant, we have first analyzed the distribution of all symptoms from the ESASr and the total score.

Table S1. The distribution analysis of all symptoms from the ESASr questionnaire.

				Skewness		Kurtosis		Shapiro-Wilk	
	**Mean**	**Median**	**SD**	**Skewness**	**SE**	**Kurtosis**	**SE**	**W**	**p**
ESASr	16.847	16.000	10.826	0.676	0.283	0.322	0.559	0.957	0.016
Pain	2.194	2.000	2.360	1.144	0.283	0.616	0.559	0.846	< .001
Tiredness	3.417	4.000	2.244	0.397	0.283	0.021	0.559	0.951	0.007
Drowsiness	2.083	2.000	2.187	1.038	0.283	1.248	0.559	0.849	< .001
Nausea	1.069	0.000	1.639	1.565	0.283	1.854	0.559	0.705	< .001
Shortness of breath	0.681	0.000	1.617	2.389	0.283	4.530	0.559	0.480	< .001
Depression	1.750	1.000	2.033	1.271	0.283	1.347	0.559	0.822	< .001
Anxiety	1.069	0.000	1.787	2.026	0.283	4.014	0.559	0.664	< .001
Wellbeing	3.181	3.000	1.779	0.043	0.283	-0.720	0.559	0.956	0.013
Other problem	1.403	0.000	2.275	2.032	0.283	3.995	0.559	0.664	< .001

I. ESASr

a) Total score

Table S2. Friedman test data for ESASr.

χ^2	df	p
15.283	2	< .001

Table S3. Pairwise Comparisons (Durbin-Conover) between the 3 moments of ESASr.

			Statistic	p
ESASr_1	-	ESASr_2	3.059	0.004
ESASr_1	-	ESASr_3	4.546	< .001
ESASr_2	-	ESASr_3	1.486	0.144

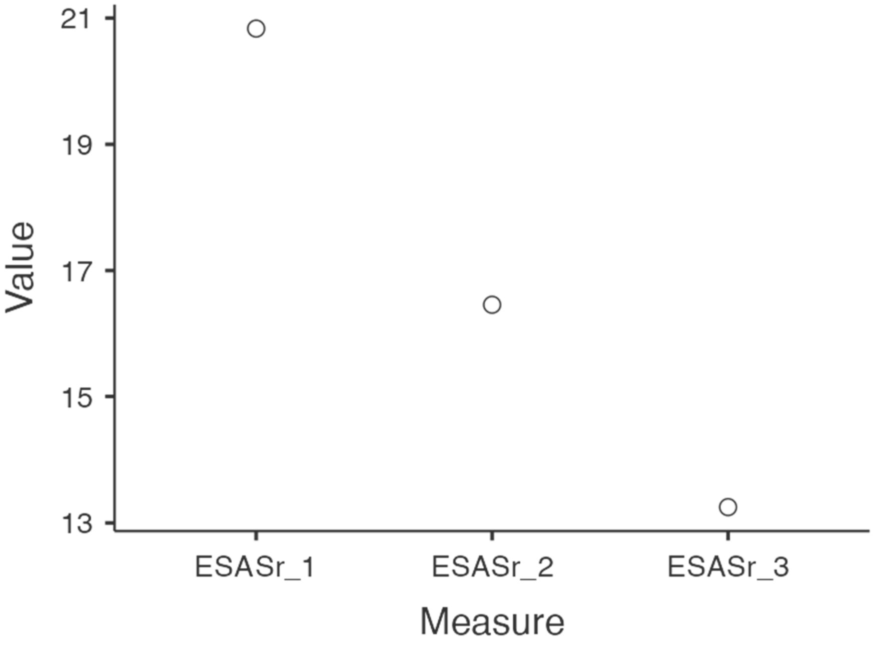

Figure S1. Descriptive plot for the three ESASr measures.

The results (and in greater detail the pairwise comparisons) reveal that there is a statistically significant difference between Time 1 and Time 3 ($p < .001$) and Time 1 and Time 2 ($p = .004$); whereas no difference exists between Time 2 and Time 3 ($p = .144$).

b) Pain

The reduction of Pain is always statistically significant. Time 2 vs Time 3 is $p = .004$; the other comparisons are significant at $p < .001$.

Table S4. Friedman test data for pain.

χ^2	df	p
31.684	2	< .001

Table S5. Pairwise Comparisons (Durbin-Conover) between the 3 moments of pain.

			Statistic	p
Pain	-	Pain (2)	6.265	< .001
Pain	-	Pain (3)	9.261	< .001
Pain (2)	-	Pain (3)	2.996	0.004

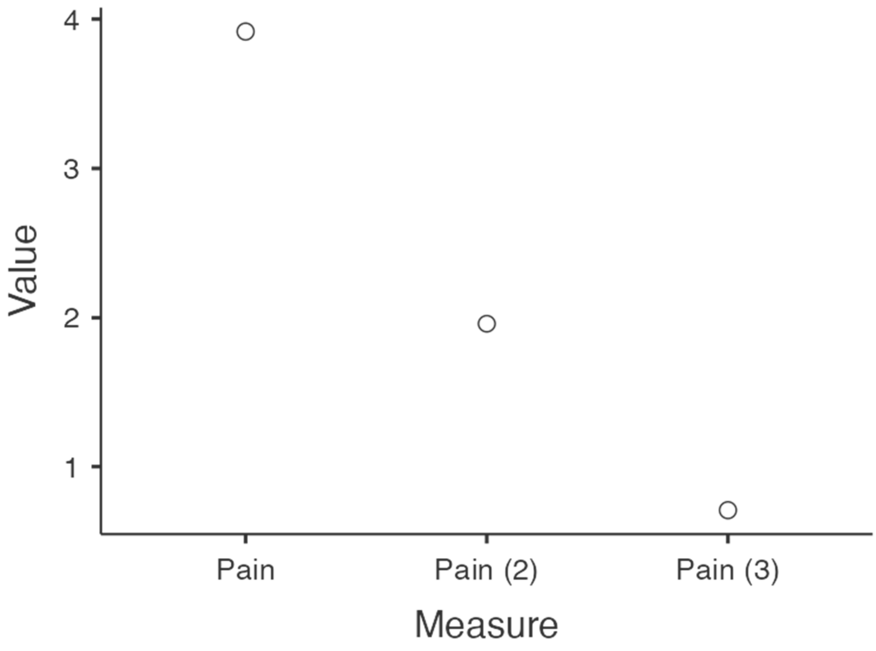

Figure S2. Descriptive plot for the three pain measures.

c) Tiredness

For tiredness we see that the reduction at Time 2 is significant (p = .006), whereas things don't improve at Time 3 (p = .436).

Table S6. Friedman test data for tiredness.

χ^2	df	p
7.760	2	0.021

Table S7. Pairwise Comparisons (Durbin-Conover) between the 3 moments of tiredness.

			Statistic	p
Tiredness	-	Tiredness (2)	2.095	0.042
Tiredness	-	Tiredness (3)	2.881	0.006
Tiredness (2)	-	Tiredness (3)	0.786	0.436

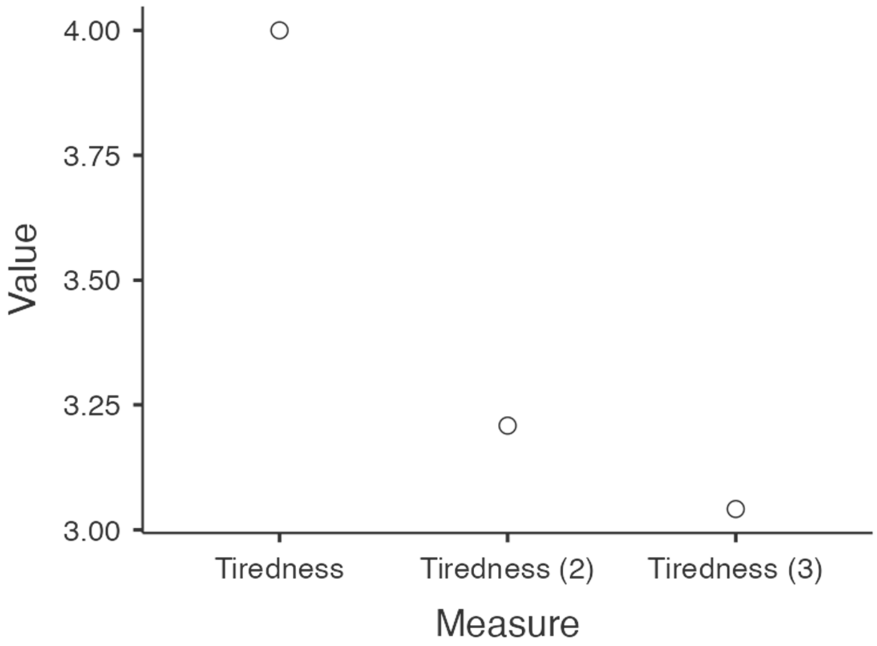

Figure S3. Descriptive plot for the three tiredness measures.

d) Drowsiness

No effect is found in reported drowsiness (p = .351).

Table S8. Friedman test data for drowsiness.

Friedman		
**χ^2**	**df**	**p**
2.094	2	0.351

Table S9. Pairwise Comparisons (Durbin-Conover) between the 3 moments of drowsiness.

			**Statistic**	**p**
Drowsiness	-	Drowsiness (2)	0.973	0.336
Drowsiness	-	Drowsiness (3)	0.442	0.660
Drowsiness (2)	-	Drowsiness (3)	1.416	0.164

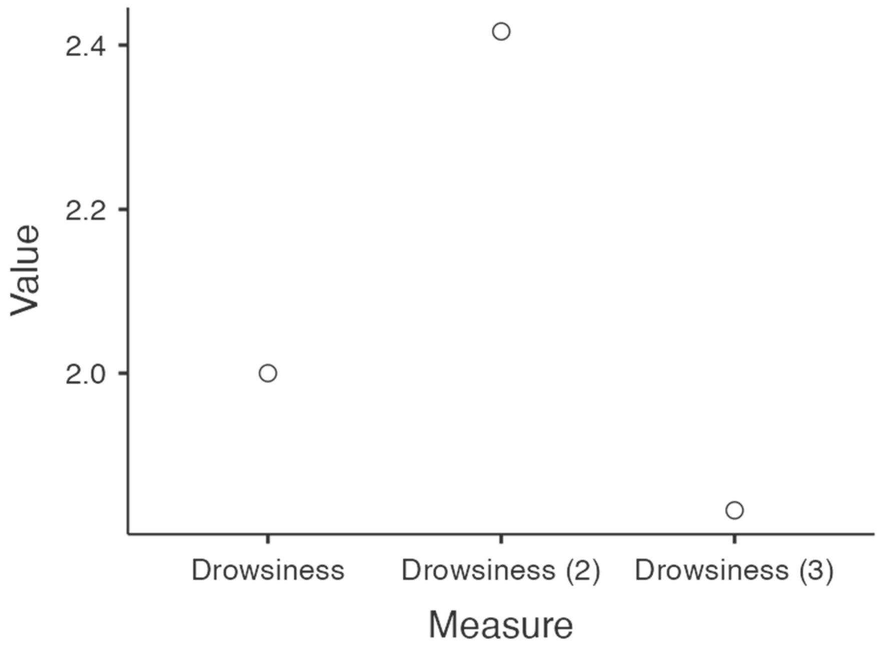

Figure S4. Descriptive plot for the three drowsiness measures.

e) Nausea

No effect is found in reported nausea (p = .133). However, if we only consider Time 1 vs Time 3, there is a marginally significant increase in nausea (p = .049).

Table S10. Friedman test data for nausea.

χ^2	df	p
4.039	2	0.133

Table S11. Pairwise Comparisons (Durbin-Conover) between the 3 moments of nausea.

			Statistic	p
Nausea	-	Nausea (2)	0.709	0.482
Nausea	-	Nausea (3)	2.026	0.049
Nausea (2)	-	Nausea (3)	1.317	0.194

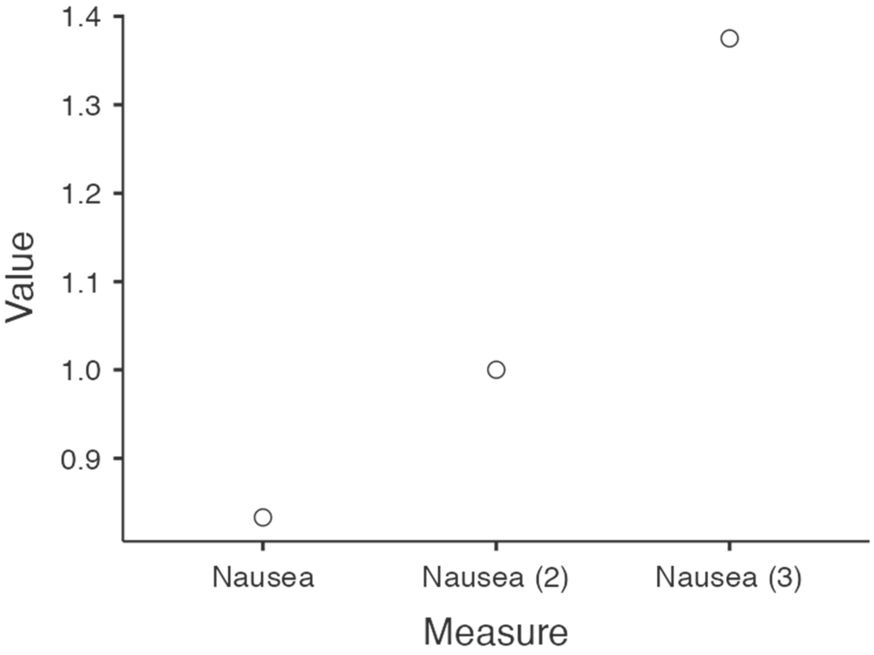

Figure S5. Descriptive plot for the three nausea measures.

f) Shortness of breath

No effect is found in reported shortness of breath (p = .355).

Table S12. Friedman test data for shortness of breath.

χ^2	df	p
2.074	2	0.355

Table S13. Pairwise Comparisons (Durbin-Conover) between the 3 moments of shortness of breath.

			Statistic	p
Shortness of breath	-	SoB (2)	0.272	0.787
Shortness of breath	-	SoB (3)	1.362	0.180
SoB (2)	-	SoB (3)	1.090	0.282

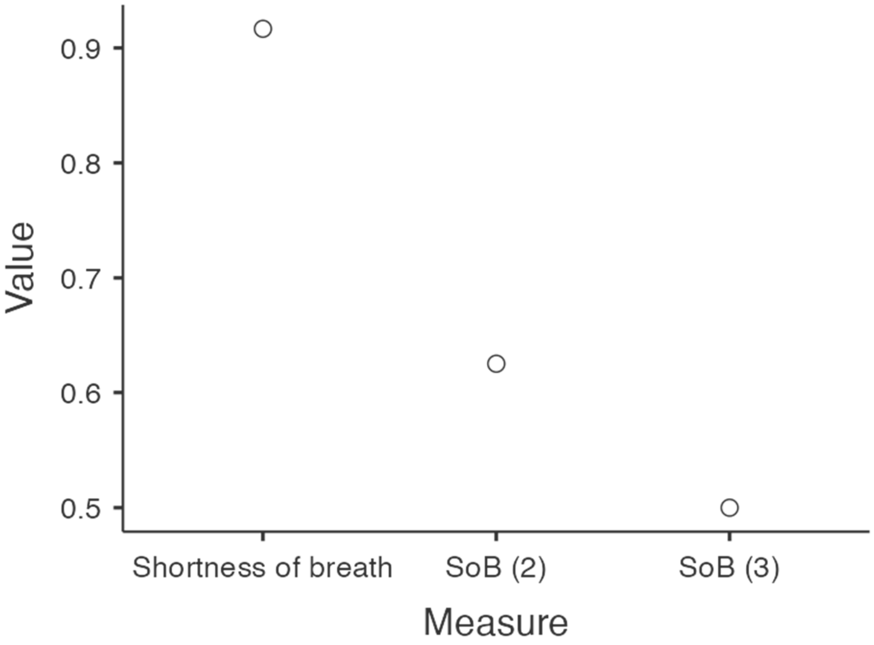

Figure S6. Descriptive plot for the three shortness of breath measures.

g) Depression

Depression scores significantly drop from Time 1 to Time 3 (p = .013), but the decreases from Time 1 to 2 (p = .376) and from Time 2 to 3 (p = .096) are not significant.

Table S14. Friedman test data for depression.

χ^2	df	p
6.290	2	0.043

Table S15. Pairwise Comparisons (Durbin-Conover) between the 3 moments of depression.

			Statistic	p
Depression	-	Depression (2)	0.894	0.376
Depression	-	Depression (3)	2.592	0.013
Depression (2)	-	Depression (3)	1.699	0.096

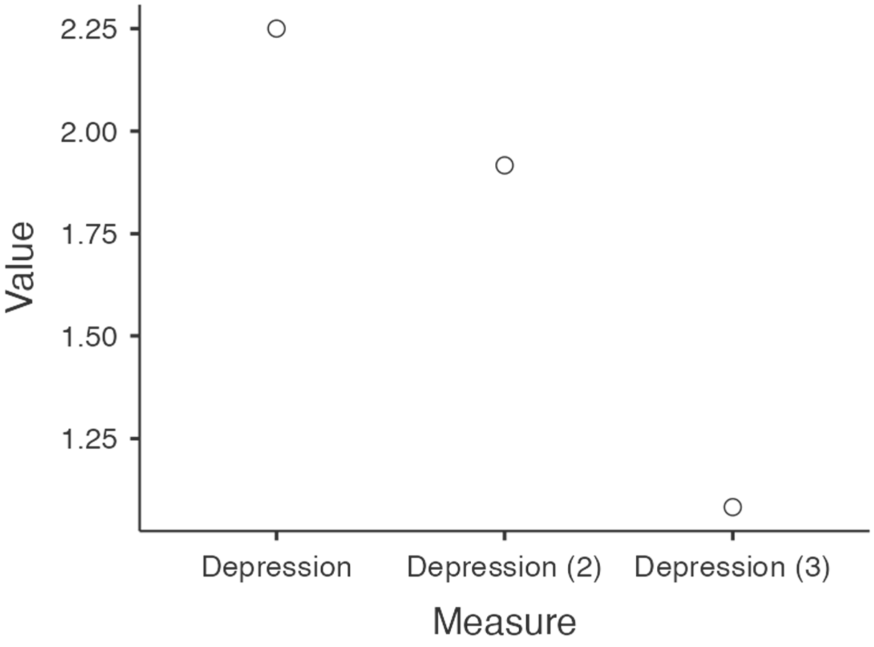

Figure S7. Descriptive plot for the depression measures.

h) Anxiety

Similarly to Depression, Anxiety scores significantly drop from Time 1 to Time 3 (p = .009), but the decrements from Time 1 to 2 (p = .102) and from Time 2 to 3 (p = .286) are not significant.

Table S16. Friedman test data for anxiety.

χ^2	df	p
6.862	2	0.032

Table S17. Pairwise Comparisons (Durbin-Conover) between the 3 moments of anxiety.

			Statistic	p
Anxiety	-	Anxiety (2)	1.669	0.102
Anxiety	-	Anxiety (3)	2.749	0.009
Anxiety (2)	-	Anxiety (3)	1.080	0.286

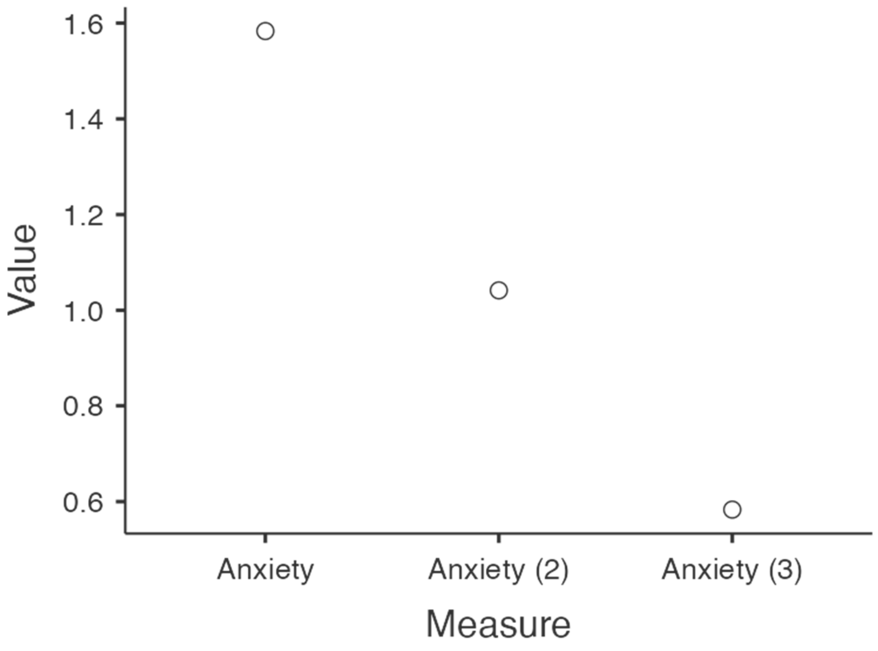

Figure S8. Descriptive plot for the anxiety measures.

i) Wellbeing

Wellbeing scores drop significantly from Time 1 to Times 2 (p = .031) and 3 (p = .013). No difference exists between Time 2 and 3 (p = .724).

Table S18. Friedman test data for wellbeing.

χ^2	df	p
6.958	2	0.031

Table S19. Pairwise Comparisons (Durbin-Conover) between the 3 moments of wellbeing.

			Statistic	p
Wellbeing	-	Wellbeing (2)	2.221	0.031
Wellbeing	-	Wellbeing (3)	2.576	0.013
Wellbeing (2)	-	Wellbeing (3)	0.355	0.724

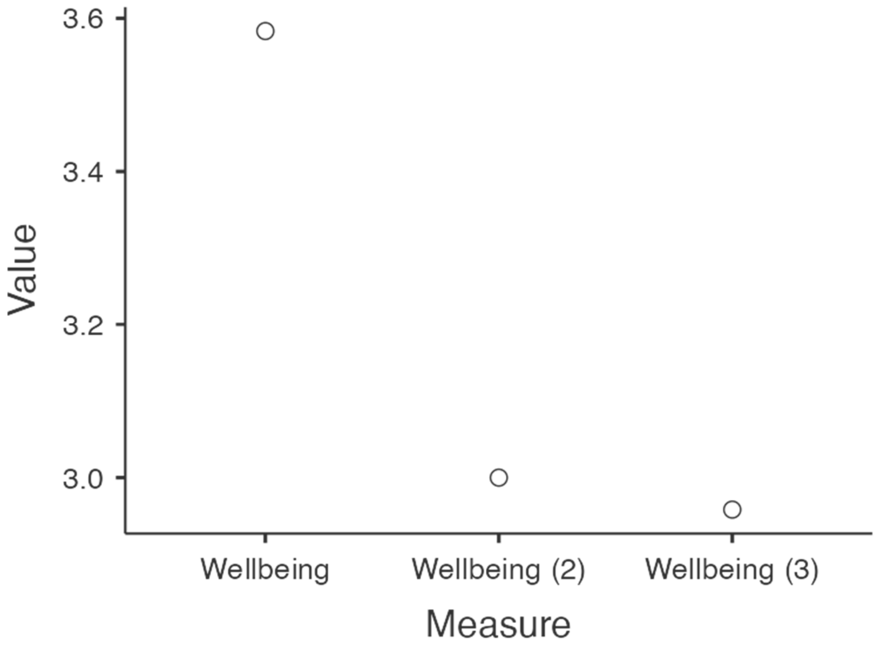

Figure S9. Descriptive plot for the wellbeing measures.

j) Other problems

No effect is found in the other problems score (p = .436).

Table S20. Friedman test data for other problems.

χ^2	df	p
1.661	2	0.436

Table S21. Pairwise Comparisons (Durbin-Conover) between the 3 moments of other problems.

			Statistic	p
Other problems	-	OP (2)	1.192	0.239
Other problems	-	OP (3)	0.183	0.855
OP (2)	-	OP (3)	1.009	0.318

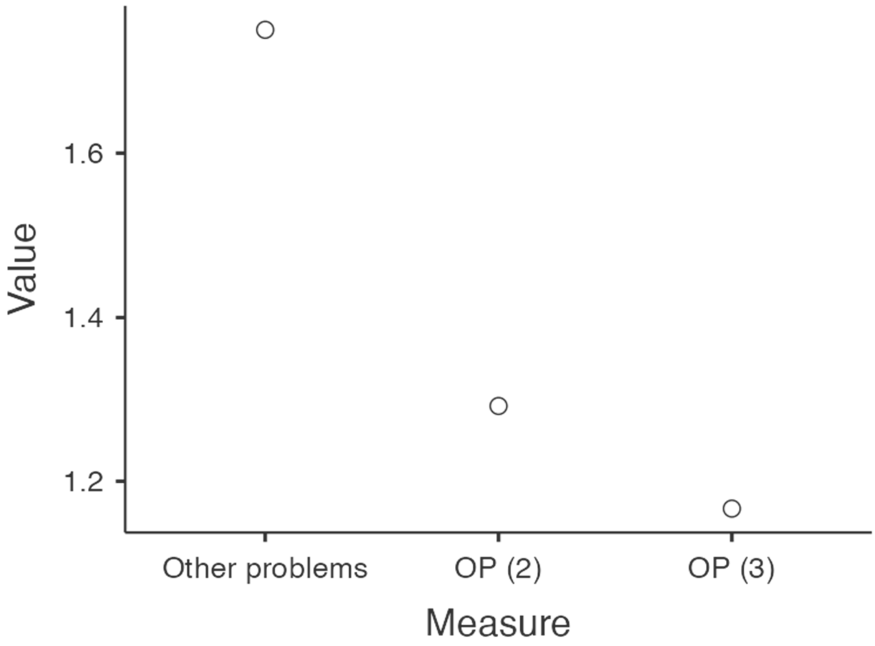

Figure S10. Descriptive plot for the wellbeing measures.

The arrangement of data from the ESASr questionnaire in the form of "Boxplot" graphs (Figures S11-S20) displays the distribution of a continuous variable. The box represents the interquartile range (IQR), which contains 50% of the data. The line inside the box represents the median. The line perpendicular to the time variable encompasses all values and extends to the maximum and minimum values - which are not considered outliers. The latter are shown as individual points. Additionally, a black square is used to indicate the mean of the data.

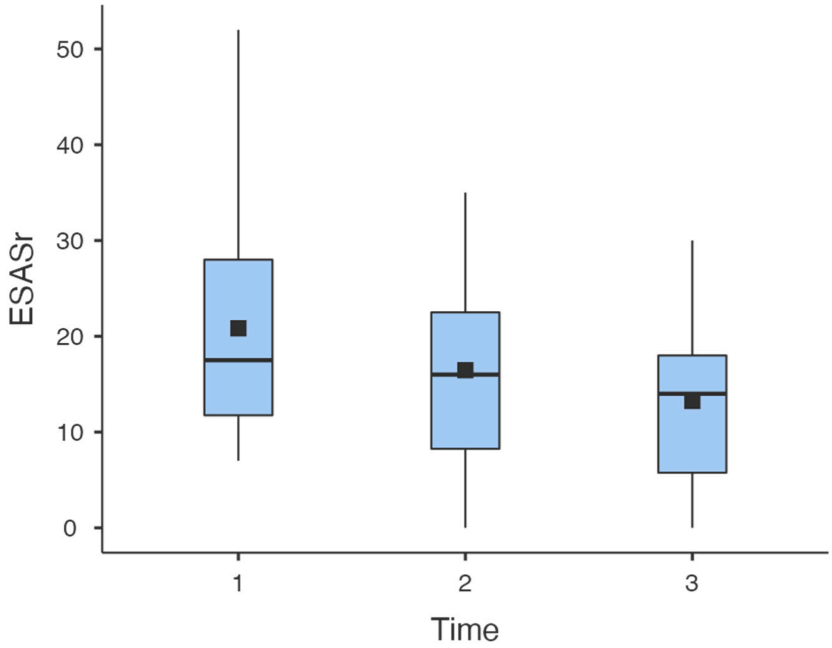

Figure S11. Boxplot graph for ESASr score.

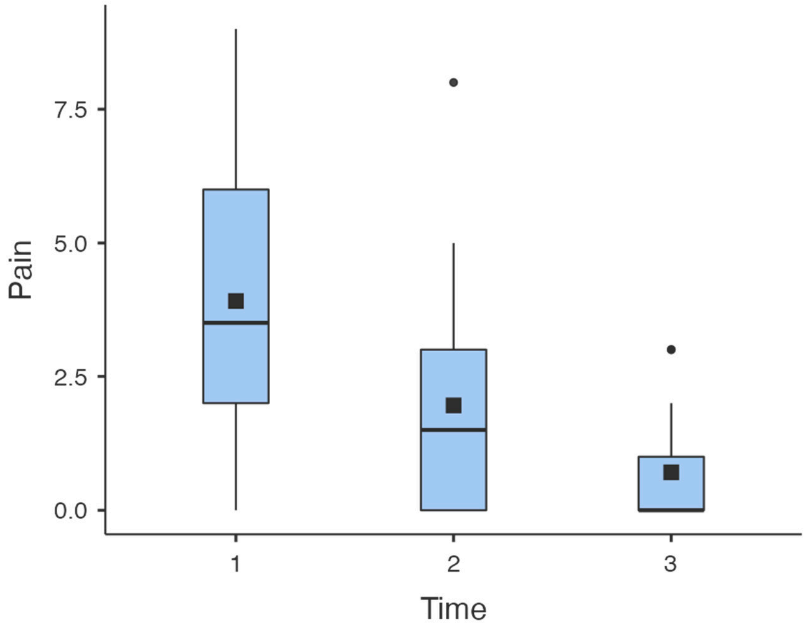

Figure S12. Boxplot graph for pain score.

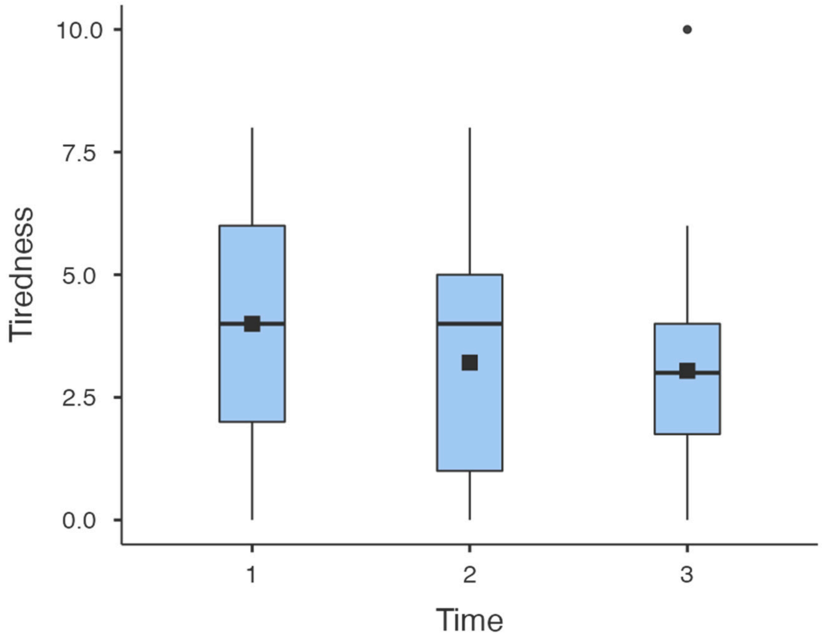

Figure S13. Boxplot graph for tiredness score.

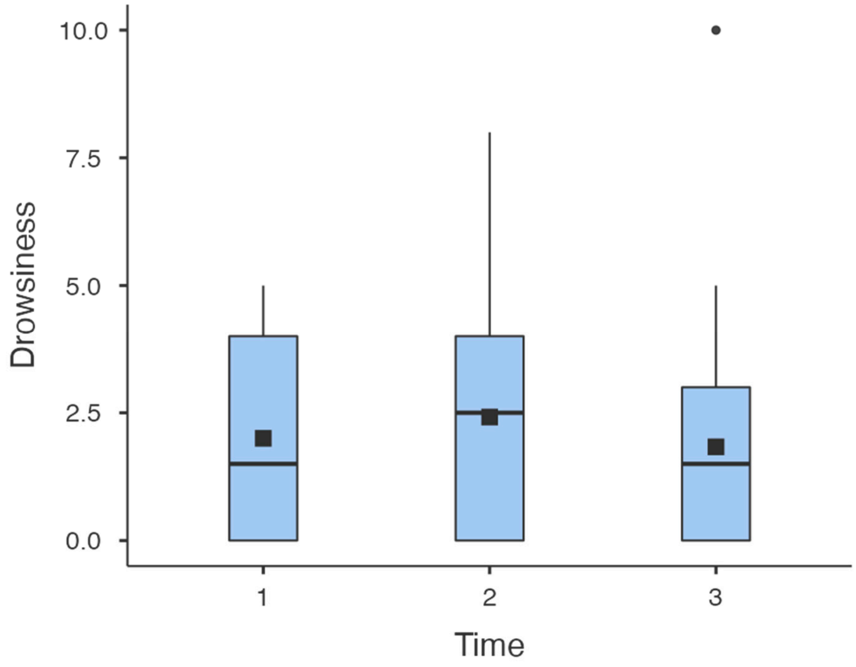

Figure S14. Boxplot graph for drowsiness score.

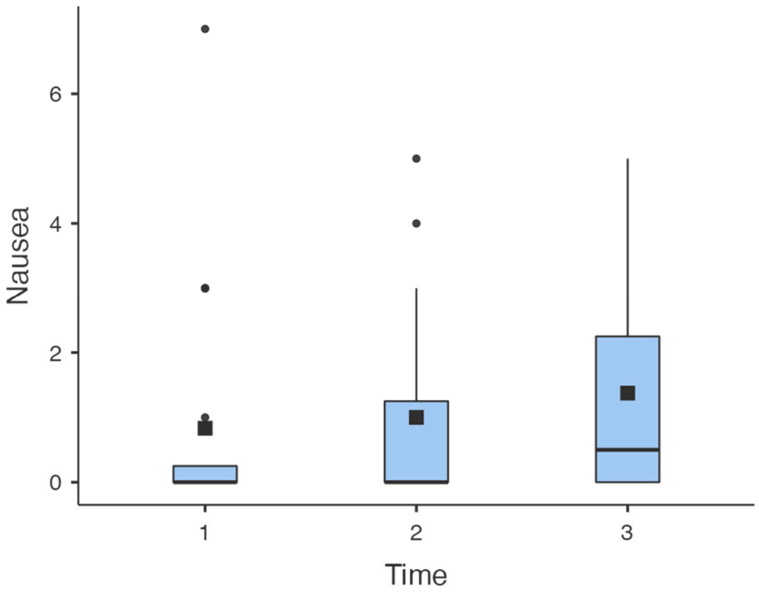

Figure S15. Boxplot graph for nausea score.

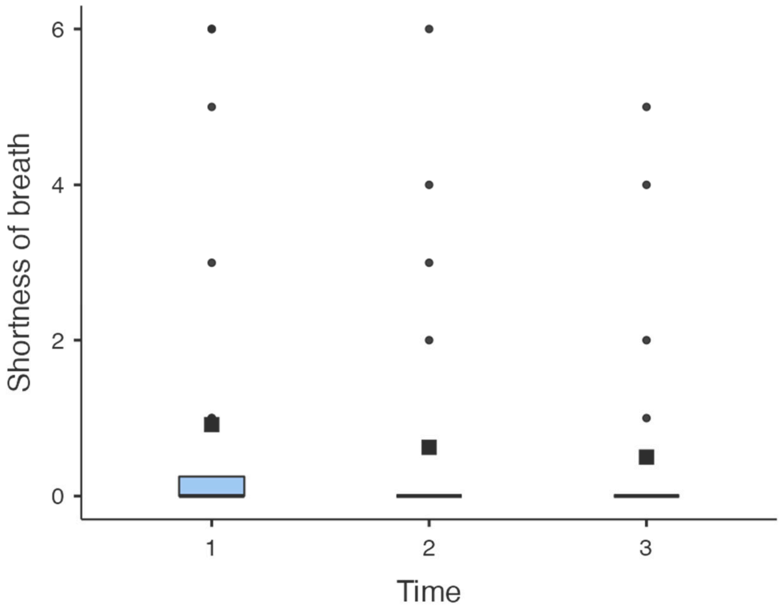

Figure S16. Boxplot graph for shortness of breath score.

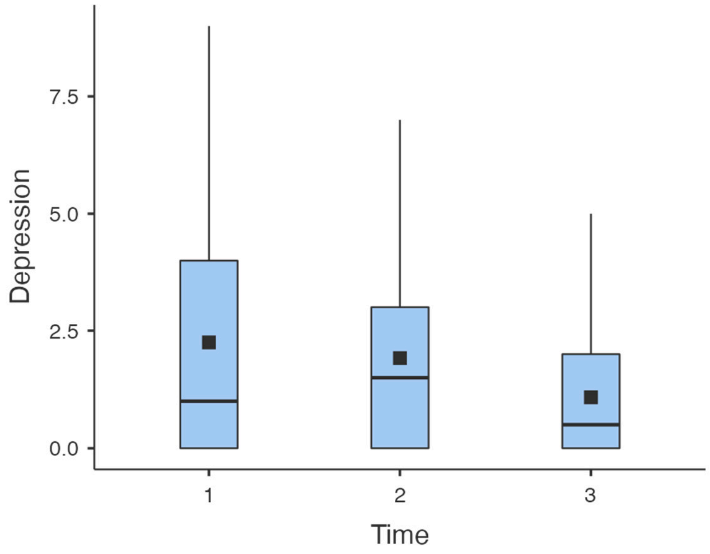

Figure S17. Boxplot graph for depression score.

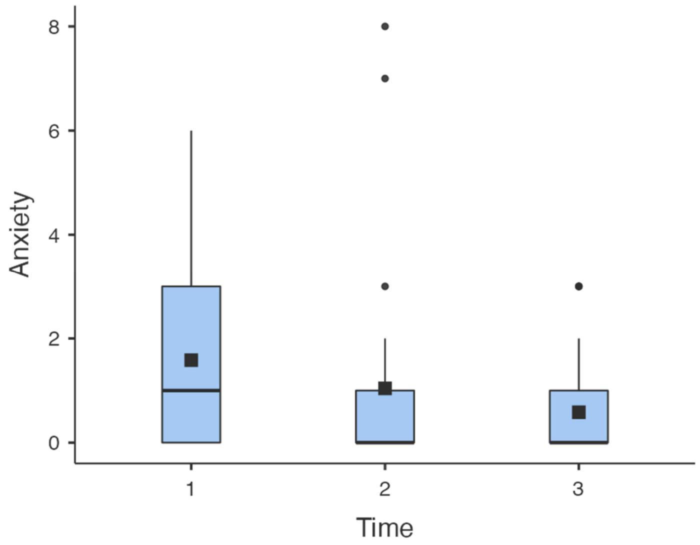

Figure S18. Boxplot graph for anxiety score.

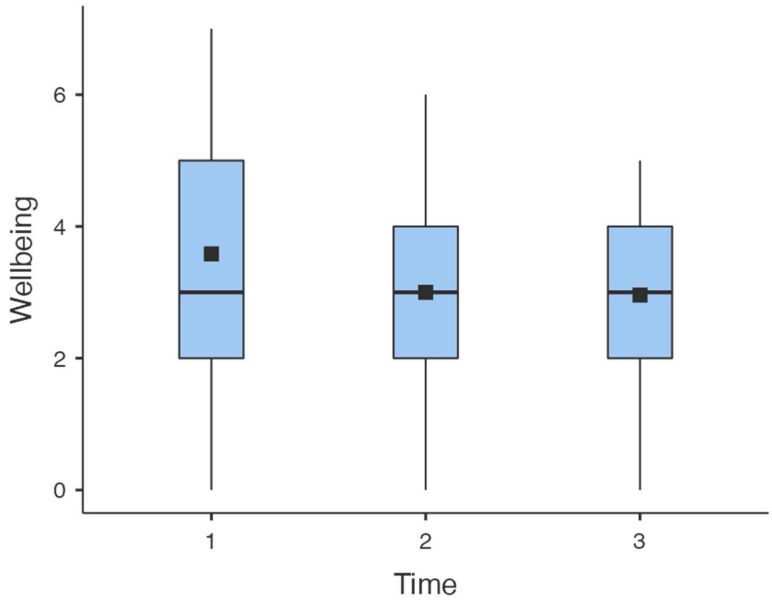

Figure S19. Boxplot graph for wellbeing score.

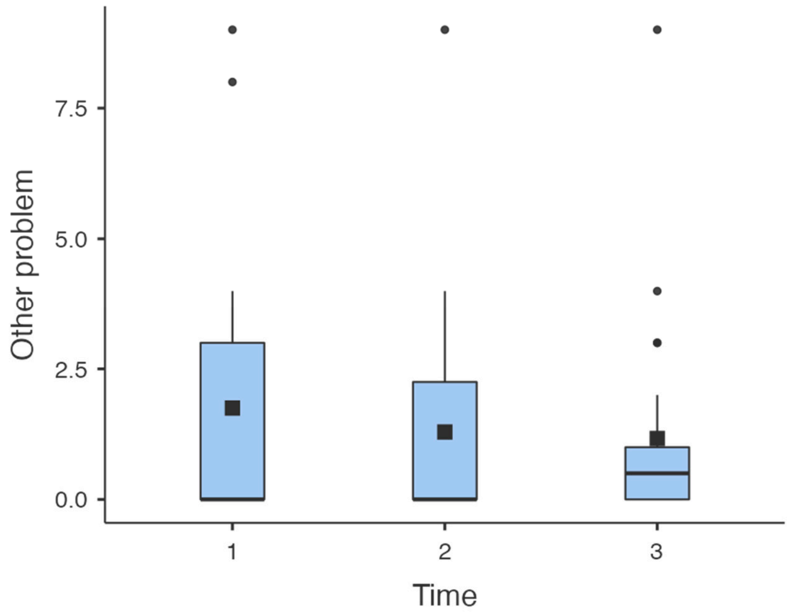

Figure S20. Boxplot graph for other problem score.

Table S22. The distribution analysis of all symptoms from the Brief Pain Inventory questionnaire.

Descriptives

				Skewness		Kurtosis		Shapiro-Wilk	
	**Mean**	**Median**	**SD**	**Skewness**	**SE**	**Kurtosis**	**SE**	**W**	**p**
General Activity	2.722	2.000	2.728	0.780	0.283	-0.344	0.559	0.876	< .001
Mood	2.931	2.500	2.724	0.561	0.283	-0.799	0.559	0.892	< .001
Walking ability	1.944	0.000	2.742	0.971	0.283	-0.673	0.559	0.710	< .001
Normal work	2.375	1.500	2.971	1.131	0.283	0.115	0.559	0.788	< .001
Relations with other people	1.292	0.000	2.146	1.826	0.283	2.793	0.559	0.671	< .001
Sleep	2.028	1.000	2.461	0.953	0.283	-0.254	0.559	0.801	< .001
Enjoyment of life	2.278	1.000	2.854	1.311	0.283	0.946	0.559	0.786	< .001

II. Brief pain inventory

a) General activity

The general activity score decreases significantly from Time 1 to Time 2 ($p = .002$), but not from Time 2 to 3 ($p = .243$). Obviously, the difference between Time 1 and 3 is significant ($p < .001$).

Table S23. Friedman test data for general activity.

χ^2	df	p
15.083	2	< .001

Table S24. Pairwise Comparisons (Durbin-Conover) between the 3 moments of general activity.

			**Statistic**	**p**
Gen. Activity	-	Gen. Activity (2)	3.251	0.002
Gen. Activity	-	Gen. Activity (3)	4.433	< .001
Gen. Activity (2)	-	Gen. Activity (3)	1.182	0.243

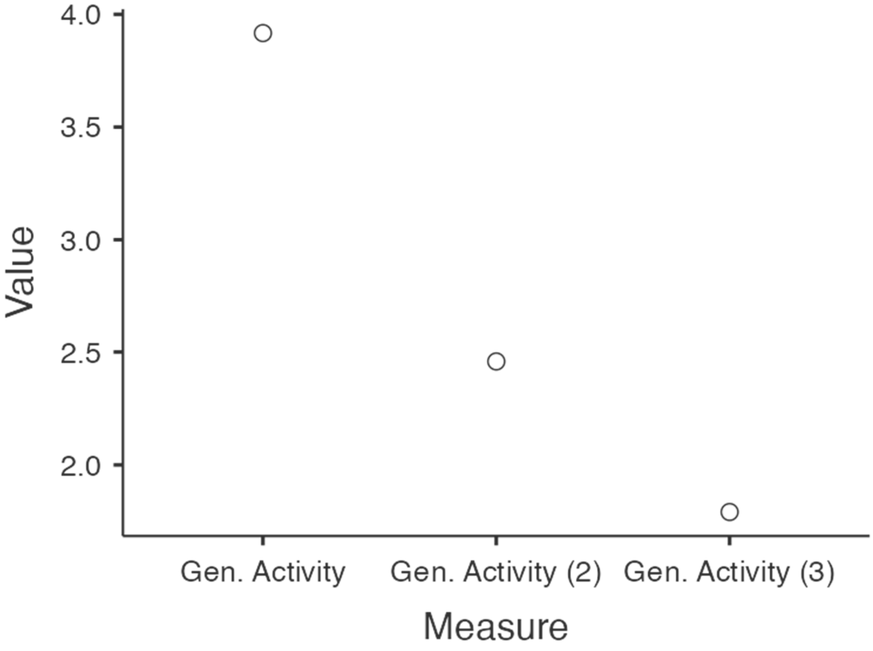

Figure S21. Descriptive plot for the general activity measures.

b) Mood

The same thing applies for mood. Its score decreases significantly from Time 1 to Time 2 ($p < .001$), but not from Time 2 to 3 ($p = .065$). Obviously, the difference between Time 1 and 3 is significant ($p < .001$).

Table S25. Friedman test data for mood.

χ^2	**df**	**p**
22.843	2	< .001

Table S26. Pairwise Comparisons (Durbin-Conover) between the 3 moments of mood.

	**Mean**	**Median**
Mood	4.458	4.000
Mood (2)	2.792	3.000
Mood (3)	1.542	0.500

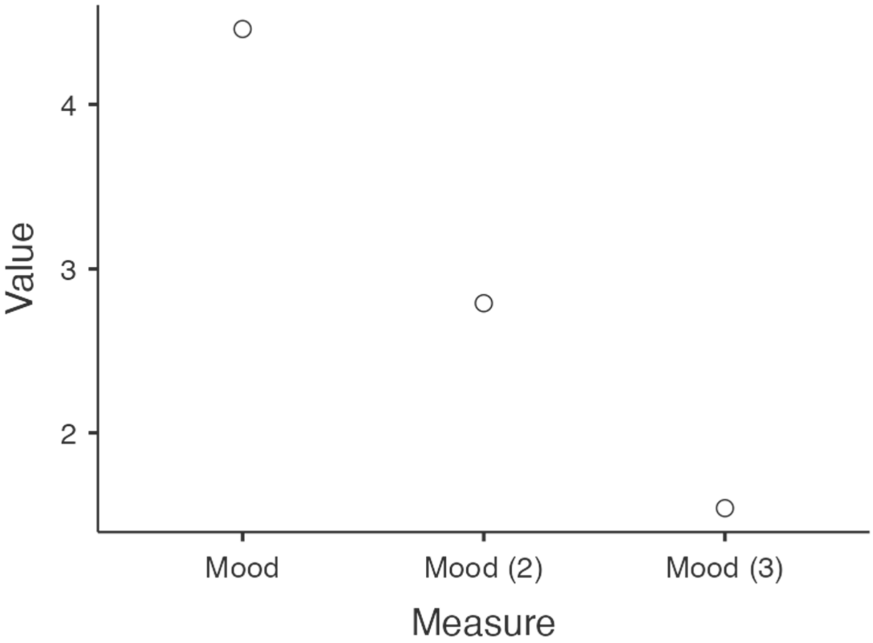

Figure S22. Descriptive plot for the mood measures.

c) Walking ability

No difference is found in walking ability ($p = .323$).

Table S27. Friedman test data for walking ability.

χ^2	df	p
2.261	2	0.323

Table S28. Pairwise Comparisons (Durbin-Conover) between the 3 moments of walking ability.

			Statistic	p
walking ability	-	walking ability (2)	1.046	0.301
walking ability	-	walking ability (3)	1.464	0.150
walking ability (2)	-	walking ability (3)	0.418	0.678

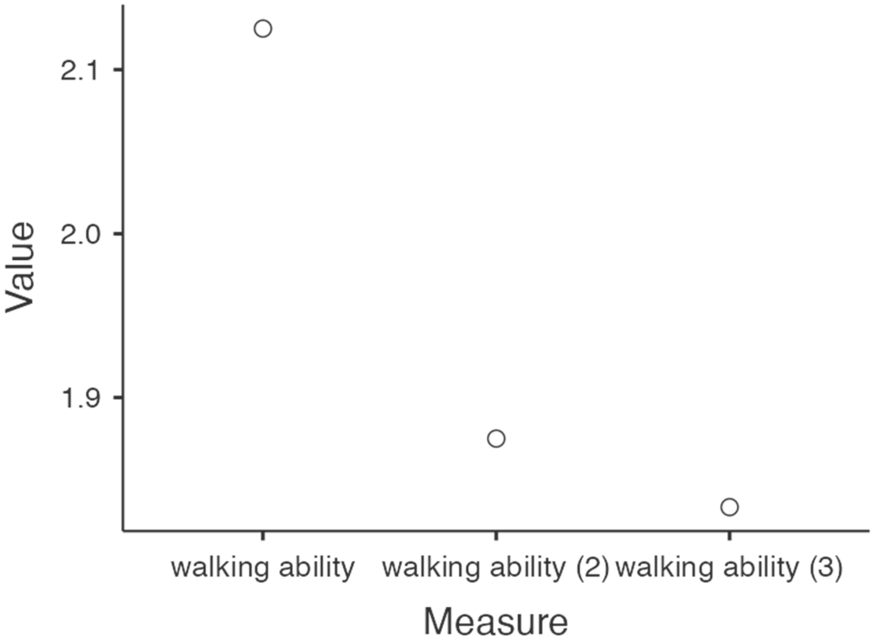

Figure S23. Descriptive plot for the walking ability measures.

d) Normal work

The pattern of normal work is identical to that observed for mood and general activity.

Table S29. Friedman test data for normal work.

χ^2	df	p
7.412	2	0.025

Table S30. Pairwise Comparisons (Durbin-Conover) between the 3 moments of normal work.

			Statistic	p
Normal work	-	Normal work (2)	2.191	0.034
Normal work	-	Normal work (3)	2.739	0.009
Normal work (2)	-	Normal work (3)	0.548	0.587

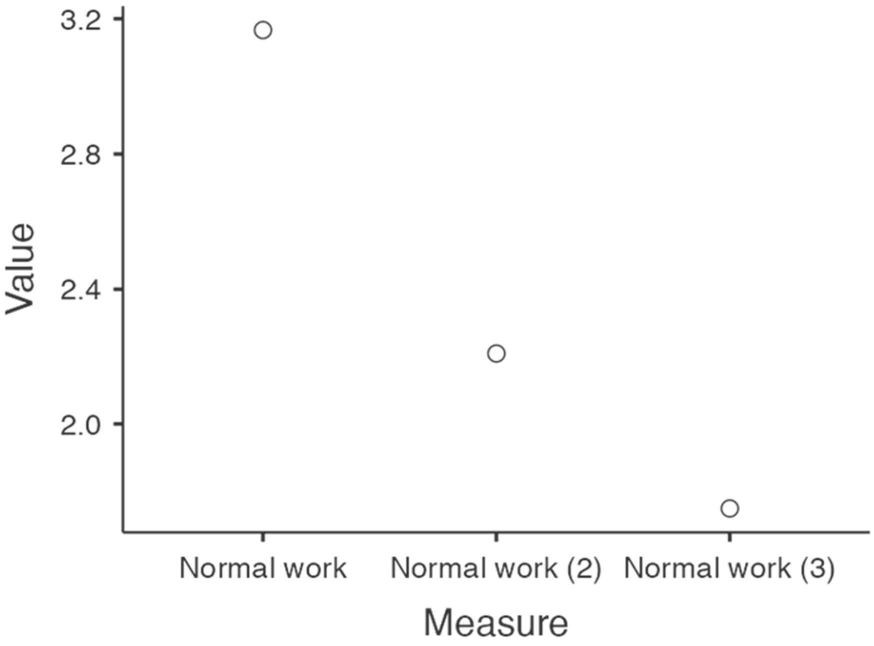

Figure S24. Descriptive plot for the normal work measures.

e) Relations with other people

No difference is found for relations with other people (p = .152).

Table S31. Friedman test data for relations with other people.

χ^2	df	p
3.774	2	0.152

Table S32. Pairwise Comparisons (Durbin-Conover) between the 3 moments of relations with other people.

			Statistic	p
relations	-	relations (2)	0.991	0.327
relations	-	relations (3)	1.981	0.054
relations (2)	-	relations (3)	0.991	0.327

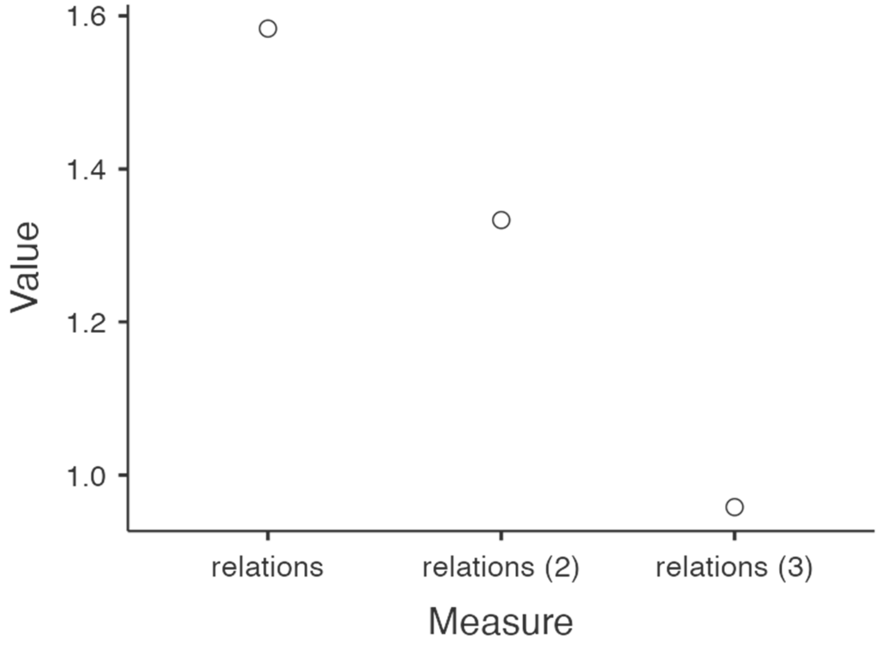

Figure S25. Descriptive plot for the relations with other people.

f) Sleep

A similar pattern emerges with mood, general activity, and normal work. Namely, Time 1 and Time 2 differ between them (p = .002), but Time 2 and 3 do not (p = .853).

Table S33. Friedman test data for sleep.

χ^2	df	p
11.863	2	0.003

Table S34. Pairwise Comparisons (Durbin-Conover) between the 3 moments of sleep.

			Statistic	p
sleep	-	sleep (2)	3.268	0.002
sleep	-	sleep (3)	3.455	0.001
sleep (2)	-	sleep (3)	0.187	0.853

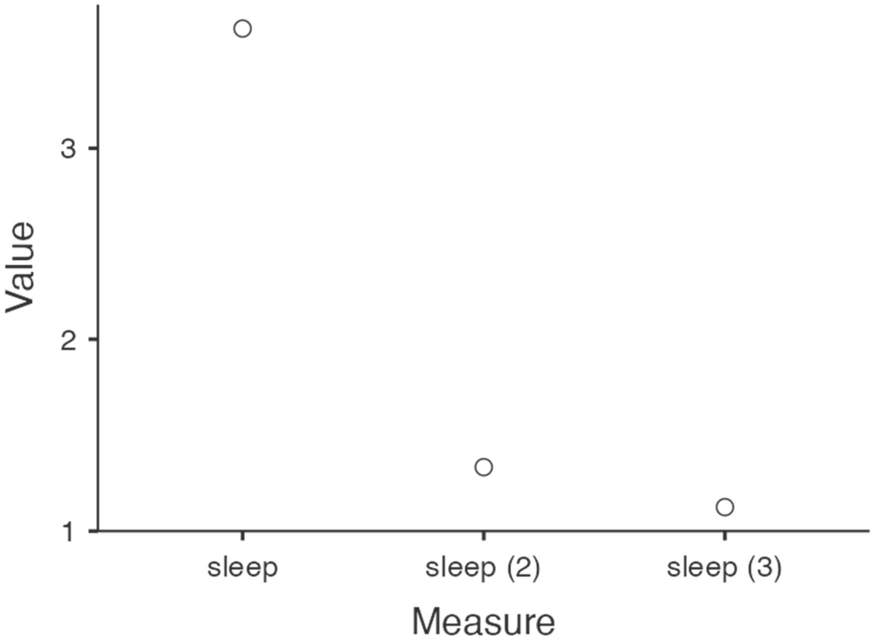

Figure S26. Descriptive plot for sleep.

g) Enjoyment of life

Regarding enjoyment of life, the only statistically significant difference is that between Time 1 and Time 3 (p = .006).

Table S35. Friedman test data for enjoyment of life.

χ^2	df	p
7.400	2	0.025

Table S36. Pairwise Comparisons (Durbin-Conover) between the 3 moments of enjoyment of life.

			Statistic	p
enjoyment of life	-	enjoyment (2)	1.169	0.248
enjoyment of life	-	enjoyment (3)	2.879	0.006
enjoyment (2)	-	enjoyment (3)	1.709	0.094

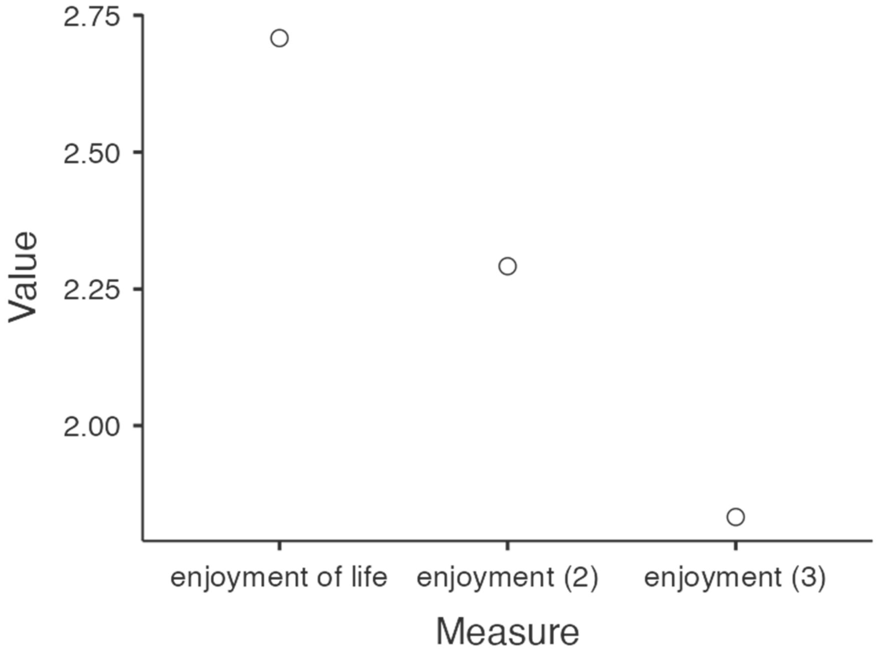

Figure S27. Descriptive plot for enjoyment of life.

The following figures (Figures S28-S34) show the distribution of continuous variables in a manner identical to that previously reported for the ESASr questionnaire (in the form of Boxplot graphs)

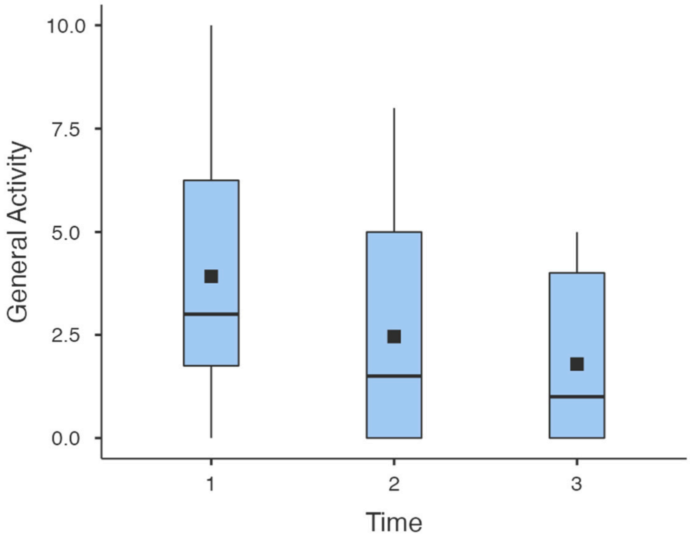

Figure S28. Boxplot graph for general activity score.

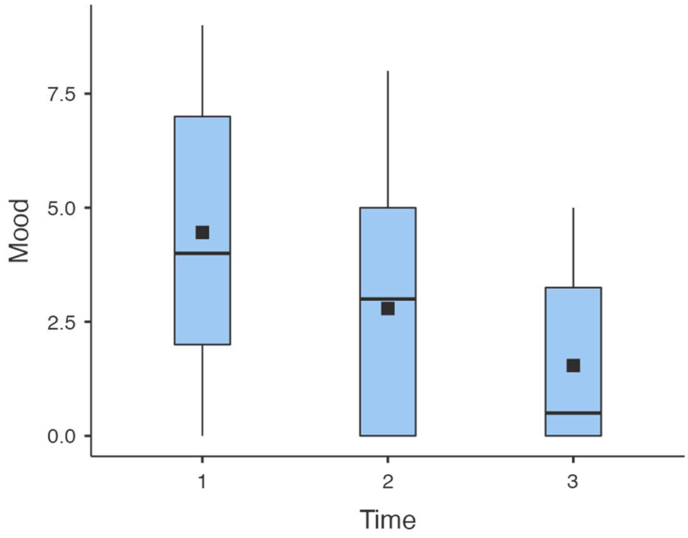

Figure S29. Boxplot graph for mood score.

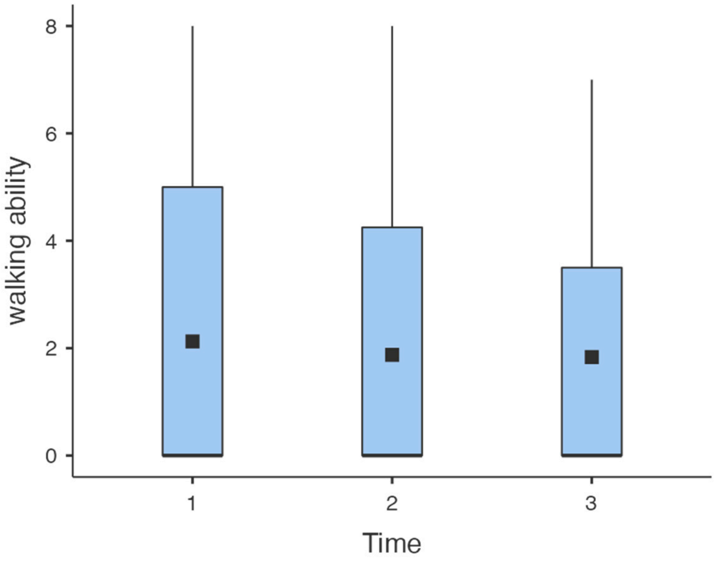

Figure S30. Boxplot graph for walking ability score.

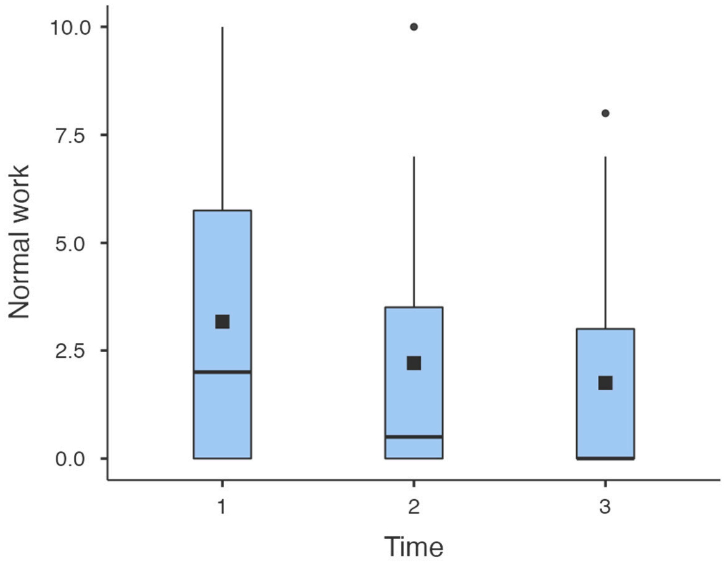

Figure S31. Boxplot graph for normal work score.

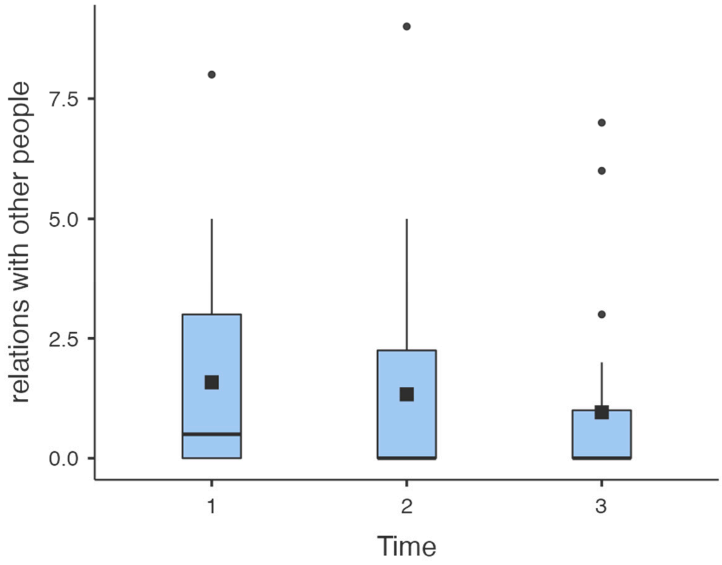

Figure S32. Boxplot graph for relations with other people score.

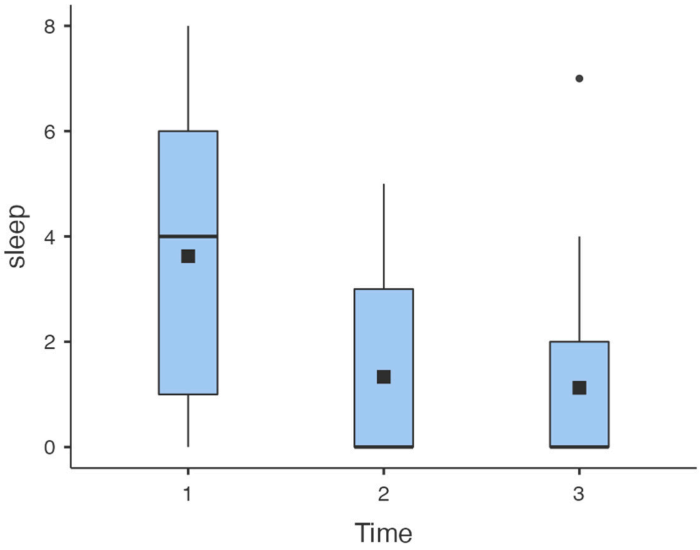

Figure S33. Boxplot graph for sleep score.

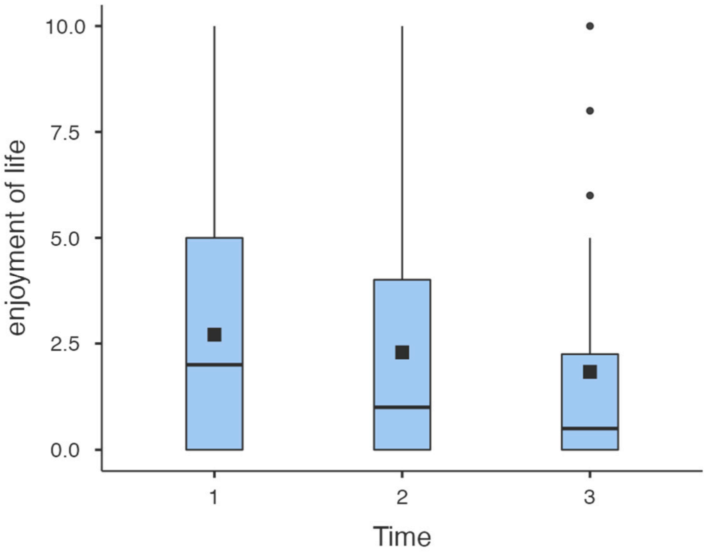

Figure S34. Boxplot graph for enjoyment of life score.
